# Overcoming Technical Complexities in Late Coronary Stent Thrombosis: A Clinical Report

**DOI:** 10.7759/cureus.47448

**Published:** 2023-10-22

**Authors:** Ben Brahim Walid, Lahjouji Reda, Choho Zakaria, Benmessaoud Fatima Azzahra, Latifa Oukerraj, Mohamed Cherti

**Affiliations:** 1 Department of Cardiology B, Centre Hospitalo-Universitaire (CHU) Ibn Sina, Rabat, MAR; 2 Department of Cardiology B, Centre Hospitalo-Universitaire (CHU) Ibn Sina, Mohammed V University, Rabat, MAR

**Keywords:** revascularization strategy, percutaneous intervention (pci), complex pci, acute coronary syndrome, late stent thrombosys

## Abstract

Complex bifurcation lesions often requiring a two-stent revascularization approach mean more metal, a higher risk of major adverse cardiovascular events, and added difficulties in the case of late complications, such as in-stent restenosis and stent thrombosis. In this article, we report a case of late stent thrombosis in a 56-year-old patient who had left main (LM) and left anterior descending (LAD) left circumflex arteries with T and small protrusion technique percutaneous intervention (PCI) one year before her admission with hemodynamic compromise and no access to urgent coronary artery bypass grafting (CABG). We discuss challenging and high-risk PCI with limited resources, and the result was satisfactory with a favorable outcome. Stent thrombosis, a critical and life-threatening complication of PCI, frequently manifests with ST-elevation myocardial infarction, carrying a high mortality risk. Known risk factors for stent thrombosis include stent underexpansion, inadequate lesion preparation, complex percutaneous procedures, and early discontinuation of dual antiplatelet therapy. The choice of revascularization strategy is crucial, particularly for patients with extensive coronary artery disease, where surgery allows for a more complete revascularization. Coronary angioplasty is a pleasing and less invasive technique, but it requires proper lesion preparation, optimization of stent deployment through intracoronary imaging, post-dilation, and, most importantly, adherence and proper use of antithrombotic treatment following guidelines and medical therapy, which remains the cornerstone of managing ischemic heart disease.

## Introduction

Bifurcation lesions comprise 15-18% [1] of all lesions treated with percutaneous coronary intervention (PCI); they are known to be technically one of the most challenging coronary interventions, requiring a higher level of skill and expertise. Particularly, bifurcation subtends large areas of myocardia, such as the distal left main coronary artery.

Complex true left main bifurcation required surgical revascularization for decades. PCI may be relevant in a selected patient. They are highly exposed to complications when the technique is not perfectly performed, especially stent thrombosis. Its management is tricky and requires knowledge of the previously used technique and endocoronary imaging.

We report a case of acute coronary syndrome in a patient with limited equipment who underwent undocumented complex PCI.

## Case presentation

A 56-year-old female, with a history of type 2 diabetes mellitus and coronary artery disease with prior intervention, was admitted to the emergency department with acute chest pain. Her symptoms started two months after the discontinuation of dual antiplatelet therapy (DAPT) due to financial reasons. The patient had a history of controlled diabetes on oral anti-diabetics and acute coronary syndrome non-ST-segment elevation myocardial infarction (ACS-NSTEMI) involving extensive coronary artery disease, including the left main (LM) coronary artery, left anterior descending (LAD) coronary artery, and left circumflex artery. The patient underwent LM PCI in another hospital.

**Figure 1:**
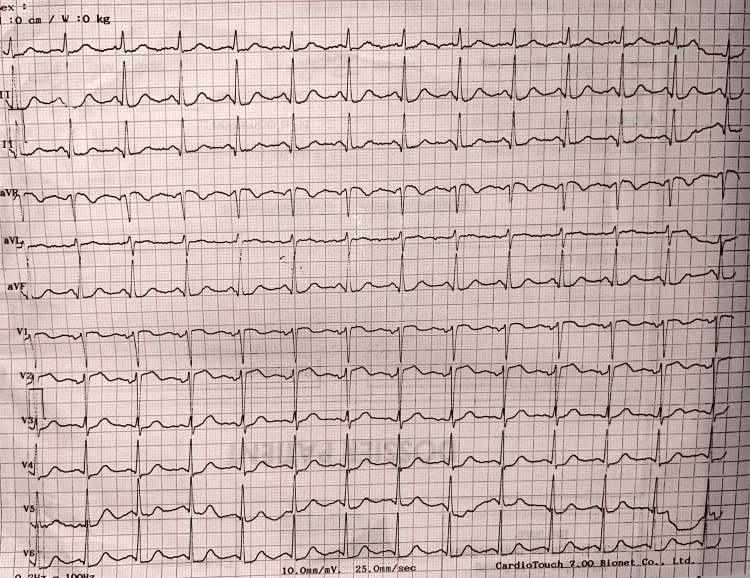
ECG showing a sinus tachycardia with ST-segment elevation in aVr- V1-V2 with diffuse ST depression in other other leads.

At admission, her blood pressure was 140/80 mmHg, her heart rate was 90 beats per minute, and her respiratory rate was 15 cycles per minute. Cardiac auscultation was unremarkable, without pulmonary crackles. Initial electrocardiography (ECG) showed a normal sinus rhythm; 1-mm ST segment elevation in aVr, V1, and V2; and ST segment depression in the other leads (Figure 1). Cardiac troponin I (cTnI) was elevated at 1,600 ng/L for a normal value in women of 16 ng/L. Transthoracic echocardiography showed a mild hypokinetic anterior and lateral wall motion with a preserved left ventricular fraction ejection at 52% by the Simpson biplane method.

**Figure 2:**
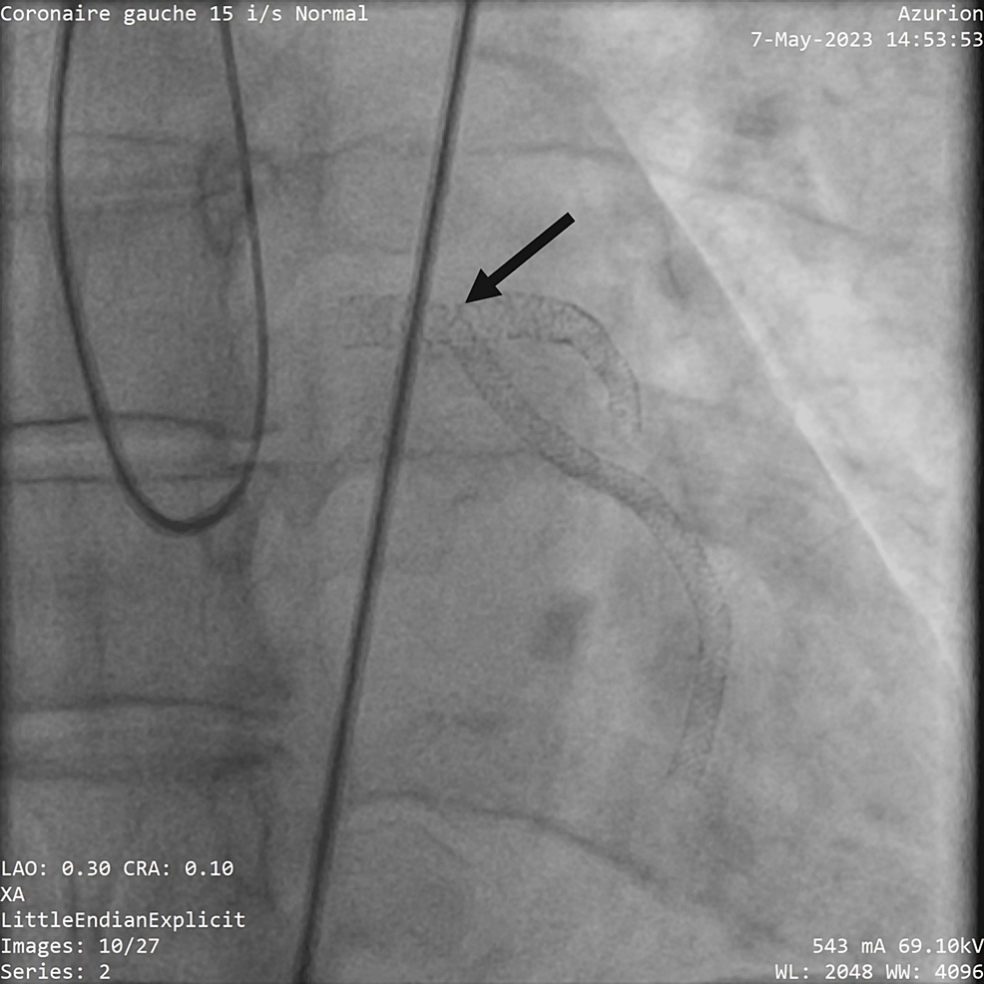
T and protrusion stenting. Image of the previous PCI showing T and small protrusion (TAP) stenting with excessive left circumflex stent protrusion (black arrow)

After the initial evaluation, the patient received a loading dose of clopidogrel 300 mg, aspirin 300 mg, and 0.6 ml enoxaparin due to the delayed invasive coronary angiography (ICA). A coronary angiogram has shown what seems to be a T and protrusion LM stenting with excessive left circumflex artery stent protrusion, with a full metal jacket in the left circumflex artery (Figure 2).

**Figure 3:**
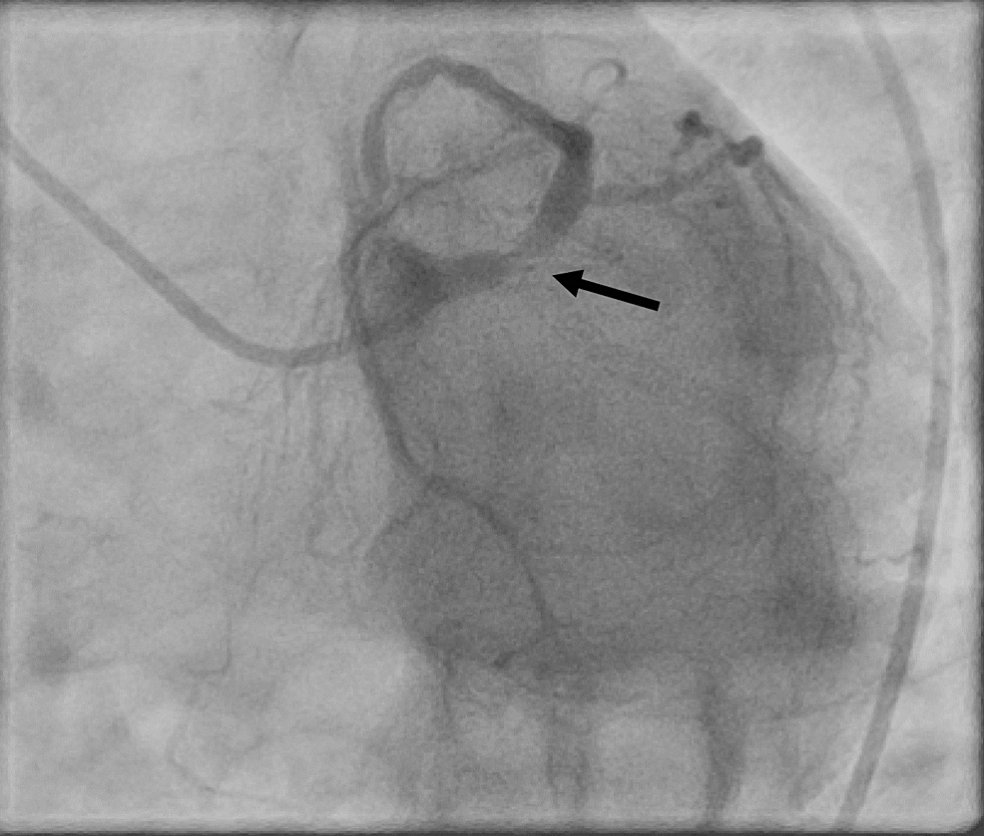
Spider view showing late stent thrombosis of the circumflex artery protruding in the left main coronary artery (black arrow).

Contrast injection revealed a late occlusion of the left circumflex artery, with a thrombotic burden in the distal LM with thrombolysis in myocardial infarction (MI) grade flow III in the LAD (Figure 3). Given the anatomical complexity, hemodynamic, and ischemic stability of the patient, urgent coronary artery bypass was considered, while the patient was put on DAPT (clopidogrel 75 mg/aspirin 75 mg), enoxaparin 0.6 ml twice a day, bisoprolol 5 mg, ramipril 5 mg, atorvastatin 20 mg, and pantoprazole 20 mg in the intensive care unit.

The patient developed, 24 hours later, multiple chest pain recurrences, syncope, and bradycardia without any electrical modifications. We decided to perform a rescue PCI with limited equipment (neither IVUS nor OCT was available).

**Figure 4:**
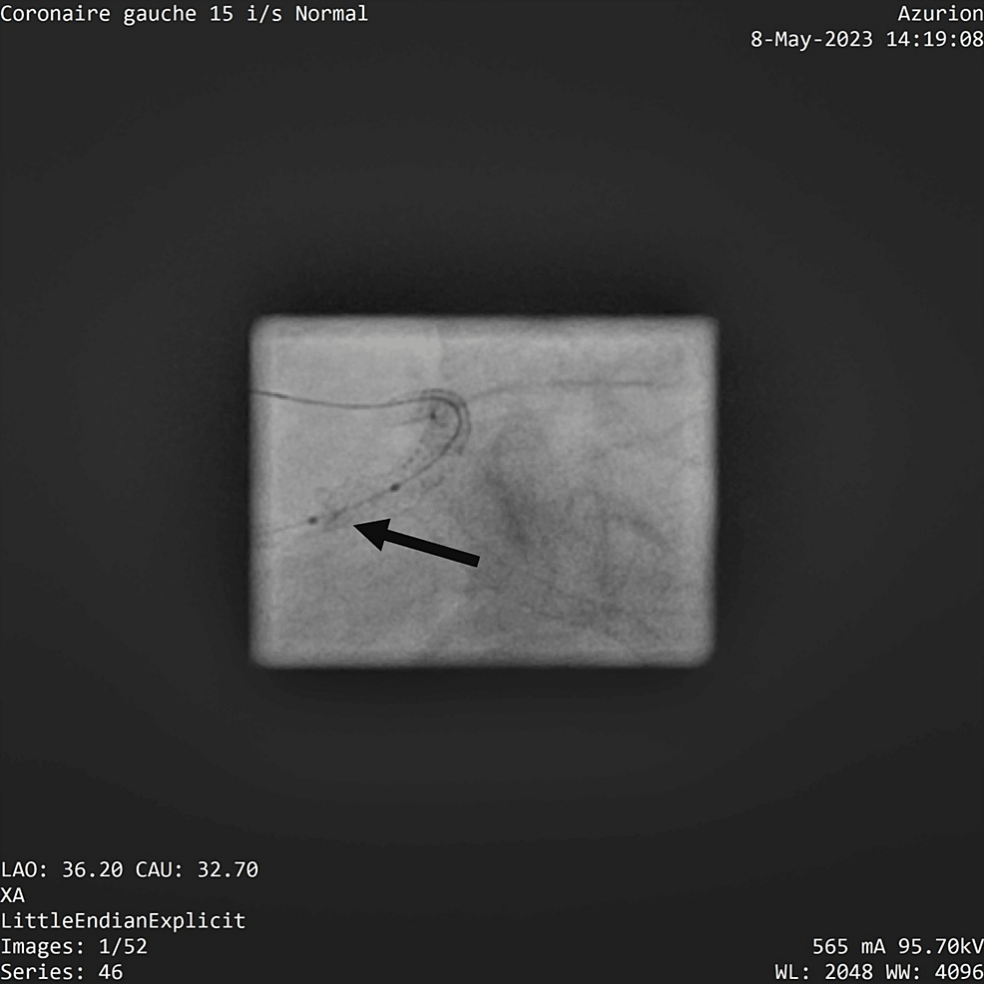
Spider view showing proximal left main struts stent deformation.

The rescue PCI was performed using a femoral access. We decided to proceed with PCI using guide catheter EBU 3.5 with easy engagement with UFH (4,800 IU intravenously). The first wire used was an intermediate support PCI guide wire, wiring the left circumflex artery with ease, and no resistance. The crossing of the second guide in the LAD was harder due to the significant protrusion of the stent from the left circumflex in the LM. Then, we pre-dilated the left circumflex with a 2 x 12 mm semi-compliant balloon and the distal left main sequentially with a 2.75x12 mm semi-compliant balloon crushing the protruding stent. The angiographic control revealed the restoration of TIMI II flow in the circumflex artery, with a heterogeneous appearance at the proximal end of the LM stent deformation of the ostial LM stent struts and hemodynamic instability. It was decided to place a stent to seal the thrombus. Advancing the stent at the LM level was impossible due to both the deformation of the stent struts and the guide wire going beneath the stent struts (Figure 4).

**Figure 5:**
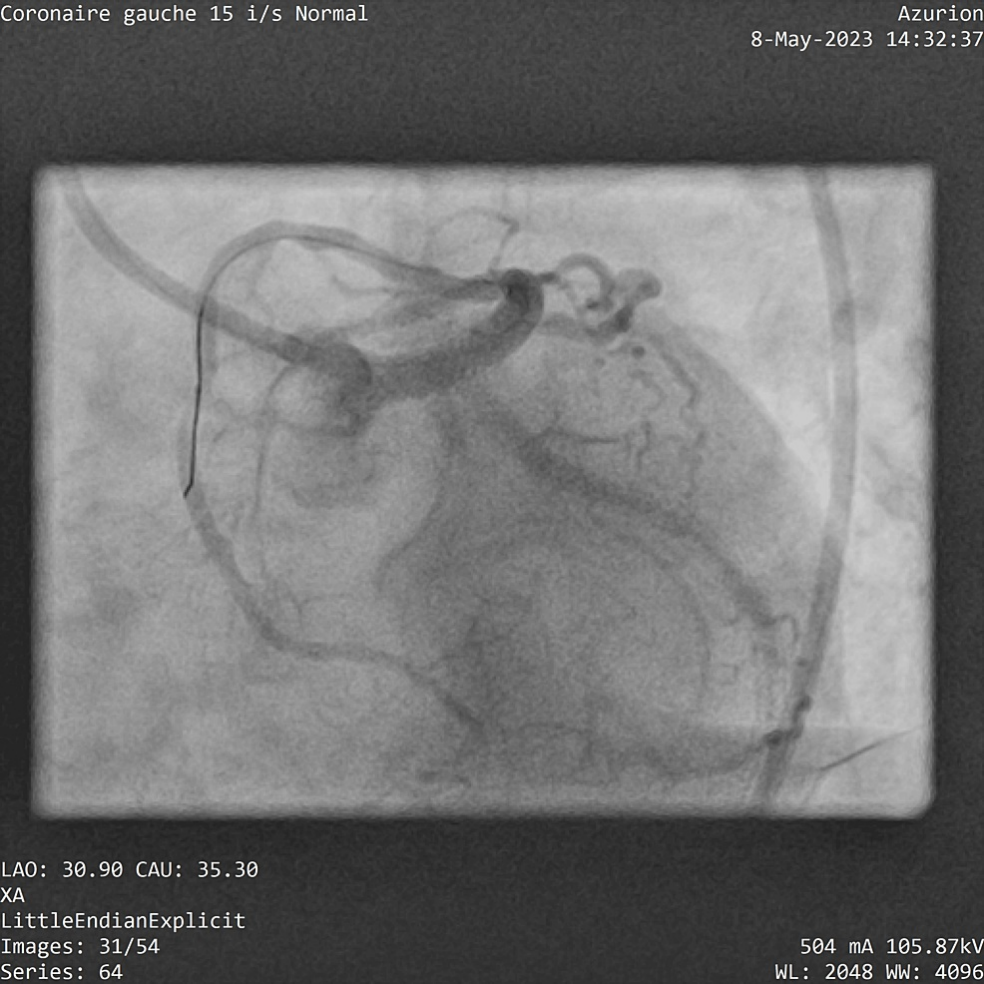
Final result with a stent using the stenting strategy of the left main and left antererior decending coronary artery.

While the hemodynamic status was deteriorating, we crushed the proximal deformed stent struts with a 3x10 mm NC balloon using the same guide wire and delivered a 3.5 x 28 mm Xience Alpine stent. Then, we proceeded to post-stenting ballooning and flaring with a 4 x 8 mm NC balloon. The final result was satisfactory (Figure 5); with the restoration of adequate hemodynamics, the patient was put on tirofiban (25 mcg/kg loading dose and then 0.15 mcg/kg for 18 h).

The patient was discharged three days later with DAPT therapy using clopidogrel 75 mg (prasugrel and ticagrelor were suggested, but the financial status of our patient did not allow it), aspirin 75 mg, bisoprolol 5 mg, atorvastatin 20 mg, ramipril 5 mg, and pantoprazole 20 mg emphasizing the importance of therapeutic adherence. At one- and three-month follow-ups, the patient did not present any functional symptoms and preserved LVFE at 55% without wall motion abnormalities.

## Discussion

PCI employing drug-eluting stents (DES) is progressively acknowledged as a legitimate revascularization alternative to CABG surgery for individuals afflicted with substantial LM artery disease [2-4]. Nonetheless, the LM artery represents the largest bifurcation within the coronary vascular system, characterized by several distinct features that may necessitate a distinct technical approach when compared to other coronary bifurcations located further downstream in the coronary tree. The myocardial territory supplied by the LM typically encompasses a substantial portion, well exceeding 50% of the overall myocardial mass. Consequently, any technical deficiencies in managing LM interventions could have a significant impact not only on procedural success but also on long-term clinical outcomes. The two main trials, EXCEL and NOBLE trials, focused on patients with left main coronary artery disease and low to intermediate SYNTAX scores (≤32), with a majority having distal left main disease and 15-29% having diabetes. In both trials, when assessing outcomes over five years, CABG showed a slight advantage over PCI. In EXCEL, CABG reduced mortality and nonprocedural MI and the need for repeat revascularizations, while in NOBLE, CABG reduced the need for repeat revascularizations and nonprocedural heart attacks, but it did not lower overall mortality. PCI appears to be a more feasible option for treating left main disease compared to complex three-vessel coronary artery disease, as indicated by the SYNTAX left main analysis. PCI is associated with a lower risk of early stroke and periprocedural MI but a higher likelihood of late MI and the need for repeat revascularization procedures [5-6]. LM stenting is challenging regarding the PCI revascularization technique, plaque preparation, adequate sizing, and stent apposition using IVUS or OCT, as well as identification of any anomaly likely to result in stent thrombosis or restenosis in cases where two stents are implanted [7].

In our case, it was a non-ST NSTEMI related to two vessels CAD, including the distal LM, in a young diabetic female patient. Given the complexity of the lesions and the patient's young age, surgical revascularization should have been the treatment of choice, but the initial intervention was performed at another facility, respecting the patient's choice. T and small protrusion seems to be the technique used for the index procedure, with excessive left circumflex stent protrusion, with little or no sensibilization to the importance of DAPT adherence, responsible for stent thrombosis. Stent thrombosis is a dramatic event usually presenting as an ST-segment elevation MI (STEMI), and it is associated with a high mortality rate varying from 25% to 40%. The incidence beyond 30 days is 0.2%-0.6% per year [8-9]. It is defined as acute if it occurs under 24 hours after PCI, sub-acute within 30 days, late between one month and one year, or very late after one year [10]. The mechanism of stent thrombosis is multifactorial, including patient-related factors, such as diabetes mellitus, chronic kidney disease, premature discontinuation or cessation of DAPT, lesion-based (diffuse CAD with long stented segments, bifurcation disease), and stent-related factors (poor stent expansion, malposition) [11]. In our case, the patient presented with a late stent thrombosis, accumulating multiple stent thrombosis-related risk factors, including diabetes mellitus, extensive coronary artery disease, multiple stent deployments, and overlapping, under-deployed circumflex arteries with excess protrusion in the LM. The particularity of our case was the guide rewiring troubleshooting of the ostial left main stent, which was deformed by previous attempts despite using a knuckled wire. While the patient was unstable, the decision was made to crush the ostial stent with a high-pressure balloon using a wire in a stent strut, perform a stent-in-stent strategy, and administer tirofiban. Given the complexity of the procedure and the acceptable result, DAPT therapy was indicated for at least 12 months following the European Society of Cardiology (ESC) guidelines, with close follow-up searching for major bleeding or ischemia.

## Conclusions

Percutaneous revascularization is a double-edged choice, finding limits in extensive coronary artery disease with complex techniques, with several stents exposed to acute or late complications and the necessity of DAPT, which early discontinuation exposes the patient to the risk of acute stent thrombosis that can be lethal. Late stent thrombosis represents a difficult and potentially life-threatening complication in clinical practice with a high mortality rate, hence the need for prevention through the selection of appropriate revascularization strategies for the right patient and treatment adherence.
